# TRIP13, identified as a hub gene of tumor progression, is the target of microRNA-4693-5p and a potential therapeutic target for colorectal cancer

**DOI:** 10.1038/s41420-022-00824-w

**Published:** 2022-01-24

**Authors:** Yan Chen, Danqi Chen, Ying Qin, Cheng Qiu, Yaoyao Zhou, Mengmeng Dai, Lulu Li, Qinsheng Sun, Yuyang Jiang

**Affiliations:** 1 School of Life Sciences, Tsinghua University, Beijing, China; 2 State Key Laboratory of Chemical Oncogenomics, Key Laboratory of Chemical Biology, Tsinghua Shenzhen International Graduate School, Shenzhen, Guangdong China; 3 Department of Gastrointestinal Surgery, Shenzhen Second People’s Hospital, Shenzhen, Guangdong China; 4 National & Local United Engineering Lab for Personalized Anti-tumor Drugs, Shenzhen Kivita Innovative Drug Discovery Institute, Tsinghua Shenzhen International Graduate School, Shenzhen, Guangdong China; 5 Shenzhen Bay Laboratory, Shenzhen, Guangdong China; 6 School of Pharmaceutical Sciences, Tsinghua University, Beijing, China

**Keywords:** Tumour biomarkers, Oncogenes

## Abstract

Colorectal cancer (CRC) is one of the digestive tract malignancies whose early symptoms are not obvious. This study aimed to identify novel targets for CRC therapy, especially early-stage CRC, by reanalyzing the publicly available GEO and TCGA databases. Thyroid hormone receptor interactor 13 (TRIP13) correlated with tumor progression and prognosis of patients after several rounds of analysis, including weighted gene correlation network analysis (WGCNA), and further chosen for experimental validation in cancer cell lines and patient samples. We identified that mRNA and protein levels of TRIP13 increased in CRC cells and tumor tissues with tumor progression. miR-4693-5p was significantly downregulated in CRC tumor tissues and bound to the 3′ untranslated region (3′UTR) of TRIP13, downregulating TRIP13 expression. DCZ0415, a small molecule inhibitor targeting TRIP13, induced anti-tumor activity in vitro and in vivo. DCZ0415 markedly suppressed CRC cell proliferation, migration, and tumor growth, promoted cell apoptosis, and resulted in the arrest of the cell cycle. Our research suggests that TRIP13 might play a crucial role in CRC progression and could be a potential target for CRC therapy.

## Introduction

Colorectal cancer (CRC) is a malignant tumor of the colon and rectum. The rising incidence of CRC is threatening people’s health and influencing the quality of life. In 2018, CRC accounted for 6.1% of global cancer incidence and 9.2% of global cancer mortality [1]. In general, when first diagnosed, the tumor stage had a significant influence on the prognosis of CRC patients [2]. CRC patients’ overall five-year survival rate gradually declined from about 90% for stage I to 70% for stage II, 58% for stage III, and less than 15% for stage IV [3]. Approximately 70–80% of CRC are sporadic tumors, which develop through the polypoid adenomas and progress into malignant forms [4, 5]. It often takes decades before final malignancies develop; hence early diagnosis and endoscopic resection of the primary tumor is essential to control CRC [6]. The molecular biomarkers of CRC tumors were tested for assistance in disease prognosis, surveillance, and treatment. CRC is considered a heterogeneous disease caused by the accumulation of genetic and epigenetic alterations [7]. Numerous genes important to CRC progression have been well-established, including KRAS, TP53, APC, PIK3CA, PPARD, and MORC2 [8–12]. However, there are no good targeted drugs for the treatment of CRC. There remains an urgent need to appraise valuable clinical entities and biomarkers for CRC therapy, especially early-stage CRC.

With the constant progression of high-throughput technologies, microarray analysis has been used to identify unknown CRC-related oncogenes and analyze gene expression in biological networks [13, 14]. The differential expression analysis considers each gene individually, and gene network analysis provides information on the numerous interactions of genes in various biological processes [15]. The weighted correlation network analysis (WGCNA) is an unsupervised classification and a data reduction method [16, 17]. The gene connectivity is interpreted as a distance, which can group genes into modules. It is assumed that the highly related genes in a module participate in a conjoint biological process. The WGCNA algorithm was used to screen out the power value in the construction of the modules and obtain the ideal soft-thresholding power. Genes were clustered into co-expressed modules according to the topological overlap matrix. WGCNA is applied for defining the gene or miRNA modules correlated with the progression of various cancers and the novel potential biomarkers related to prognosis [18, 19]. This study aimed to identify some novel targets for the therapy of CRC, primarily early-stage CRC. We hypothesized that genes played a key role in the tumor progression should be continuously dysregulated from an early stage. Then, we defined the differentially expressed genes (DEGs) between the early-stage CRC patients and healthy controls in the GSE9348 gene expression chip and used the GSE9348 DEGs to apply WGCNA in the GSE41258 data. The co-expressed modules and hub genes correlation with the tumor progression were investigated. The Hub gene, thyroid hormone receptor interactor 13 (TRIP13) whose expression increased continuously from adjacent mucosa to adenoma to early-stage to late-stage tumors was verified in the GSE117606 dataset and tested in the clinical specimens and various CRC cell lines.

There is increasing evidence that showed upregulated TRIP13 levels play a role in some tumors, including head and neck cancer, breast cancer, lung cancer, liver cancer, prostate cancer, gastric cancer, and human chronic lymphoblastic leukemia [20, 21]. TRIP13 regulates spindle assembly checkpoint by remodeling its effector Mad2 from “blocked” (active) to “open” (inactive) [22]. In addition, TRIP13 shRNA inhibits the proliferation of CRC cells and tumor growth of xenograft CRC mice [23]. However, the possible molecular mechanism of TRIP13 upregulation in CRC is unclear. Moreover, it is necessary to study the anti-tumor activity of small molecule inhibitors targeting TRIP13 to determine whether TRIP13 is a potential target for CRC therapy. Thus, the present study focused on elucidating these aspects. DCZ0415 [24], a small molecule inhibitor targeting TRIP13 was used in our study.

## Results

### Identification of DEGs and weighted correlation network construction

**Fig. 1:**
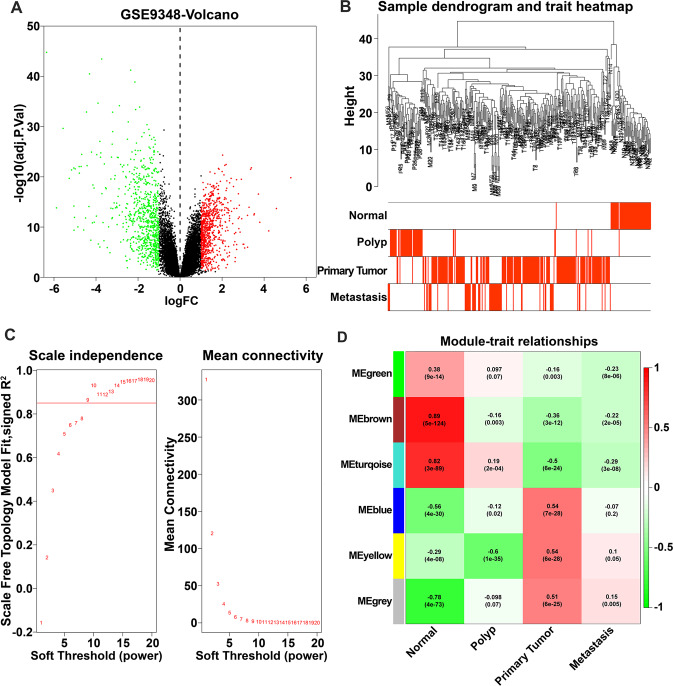
Identification of DEGs in GSE9348 dataset and the key gene modules related to tumor progression in GSE41258 dataset. **A** Volcano plots of DEGs in GSE9348 dataset. Red and green dots respectively indicated the upregulated and downregulated genes. **B** The heatmap of sample dendrogram and trait. The 1232 genes of 355 samples in GSE41258 were used to apply WGCNA. The clinical traits were normal, polyp, primary tumor, and metastasis. **C** Analysis of the scale-free fit index for various soft-thresholding power (*β*). Analysis of the mean connectivity for various soft-thresholding power. **D** Heatmap of the correlation between module eigengenes and the clinical trait of CRC. The blue module with 270 genes was most significantly positive associated with primary tumor and also negative associated with normal and polyp tissues.

The volcano plots were used to visualize all the DEGs between 70 CRC patients at an early stage and 12 healthy controls from the GSE9348 dataset. About 1595 DEGs with 813 upregulated and 782 downregulated genes were determined (Fig. 1A). Among the 1595 DEGs of the GSE9348 dataset, 1232 genes were expressed in the GSE41258 samples. These 1232 genes expression of 355 samples in GSE41258 was used to construct the scale-free network by the WGCNA package and get the potential gene modules connected with tumor progression. The heatmap of sample dendrogram and trait was constructed (Fig. 1B). The power value, which affected the scale independence and the mean connectivity of the co-expression module, was a critical parameter. The fittest power value guaranteeing the high scale independence and low mean connectivity was 9 (Fig. 1C). Six co-expression modules correlated with clinical traits, and the heatmap of module-trait relationships were constructed (Fig. 1D). The blue module with 270 genes was most significantly positively correlated with primary tumor (*r* = 0.54, *p* = 7 × 10^−^^28^) and also negatively correlated with normal (*r* = −0.56, *p* = 4 × 10^−^^30^) and polyp tissues (*r* = −0.12, *p* = 0.02). The correlation between gene significance for polyp and primary tumor and module membership in the blue module was measured (Fig. S1A, B). Meanwhile, the heatmap of connectivity degree among different modules was constructed, and the blue and yellow modules had higher adjacency values (Fig. S1C).

### Functional pathways enrichment analysis and selection of hub genes

**Fig. 2:**
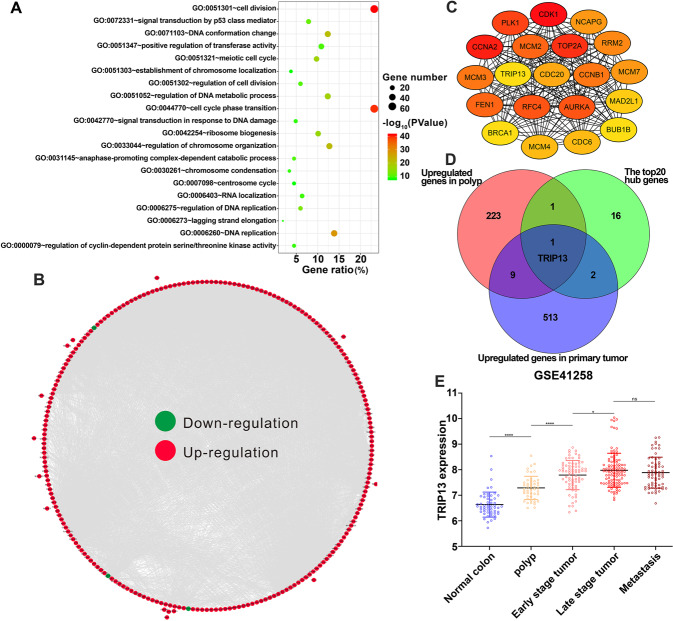
Functional pathways enrichment analysis and selection of hub genes in the blue module. **A** Heatmap of top 20 functional path enrichment analysis. **B** PPI network of the blue module genes. **C** The top 20 hub genes identified from the whole network by using the radiality algorithm of plug-in cytoHubba. **D** Venn diagram of the top 20 hub genes, the upregulated genes of polyp vs. normal tissues, and the upregulated genes of primary tumor vs. polyp tissue in GSE41258 dataset. **E** TRIP13 expression of normal, polyp, early stage tumor, late stage tumor, and metastasis tissues in GSE41258 dataset.*p < 0.05, ****p < 0.0001; *ns,* no significant difference.

The Metascape database was used to perform the GO and KEGG enrichment analysis to investigate the functional differences and the involved biological processes of 270 genes in the blue module. A heatmap was constructed according to the top 20 significant pathways and functions with a *p*-value < 0.01 (Fig. 2A). The results showed that there were six parts in the significant enrichment of biological processes: regulation of cell cycle and division, signal transduction in response to DNA damage and p53 class mediator, regulation of DNA replication and metabolic process, regulation of chromosome organization and localization, ribosome biogenesis and RNA localization, centrosome cycle. According to the correlated function pathway and constructed network, the highly enriched genes were clustered by different categories and the number of genes (Fig. S2A, B). To further explore the biological roles of the blue module genes, the STRING database was used to apply the protein−protein interaction (PPI) enrichment analysis and the network constructed by Cytoscape (Fig. 2B). Except for the independent nodes, which did not interact with others, there were 237 nodes and 5099 edges in this network. The radiality algorithm of plug-in cytoHubba was used to identify the top 20 hub genes from the whole network (Fig. 2C). A heatmap of the involved biological processes of the 20 hub genes was also constructed (Fig. S2C). Among the top 20 hub genes, only the Hormone Receptor Interactor 13 (TRIP13) expression increased continuously from normal tissue to polyp to early-stage tumor to the late-stage tumor in the GSE41258 dataset, suggesting that TRIP13 might play an essential part in tumor progression (Fig. 2D, E). Thus, TRIP13 was selected for further verification and research.

### Validation of TRIP13 expression in GSE117606 data, clinical specimens, and CRC cell lines

**Fig. 3:**
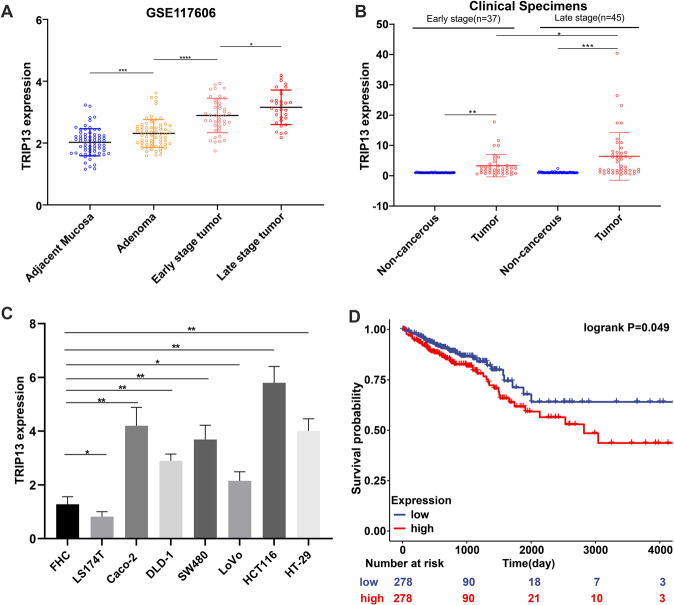
Independent validation of TRIP13 expression in GSE117606, clinical specimens, and CRC cell lines. **A** TRIP13 expression of adjacent mucosa, adenoma, early stage tumor, and late stage tumor tissues in GSE117606 dataset. **B** TRIP13 mRNA expression was drastically increased in CRC tumor. Compared to the early stage, TRIP13 mRNA levels were significantly higher in the late stage tumor. **C** TRIP13 mRNA expression was significantly increased in CRC cells compared to FHC cells. **D** TRIP13 expression was correlated with survival of CRC patients. Low expression of TRIP13 conferred a survival advantage to CRC patients. *p < 0.05, **p < 0.01, ***p < 0.001, ****p < 0.0001.

Another independent GEO gene expression profile (GSE117606) was used to validate TRIP13 expression. TRIP13 expression increased continuously from adjacent mucosa to adenoma to early-stage to late-stage tumors (Fig. 3A). TRIP13 mRNA and protein expression were tested in our clinical specimens. The TRIP13 mRNA and protein expression distinctly increased in tumor tissues of 82 CRC patients compared to non-cancerous tissues (Figs. 3B and S3). A significant association was observed between the mRNA expression of TRIP13 with the CRC tumor grade (Fig. 3B). The TRIP13 mRNA and protein expression were also significantly higher in the CRC cells, consistent with the results in tumor tissues (Figs. 3C and S4). The clinical parameters and survival data of 556 CRC patients were extracted from the TCGA database, and a Kaplan-Meier survival curve was plotted. The patients were divided into two groups according to the median value of TRIP13 expression: high and low expression separately represented by red and blue. Low TRIP13 expression conferred a survival advantage to CRC patients, suggesting that TRIP13 expression might have prognostic value (Fig. 3D).

### miR-4693-5p targeted TRIP13 through binding to its 3′UTR and decreased in CRC tumor tissues and cell lines

**Fig. 4:**
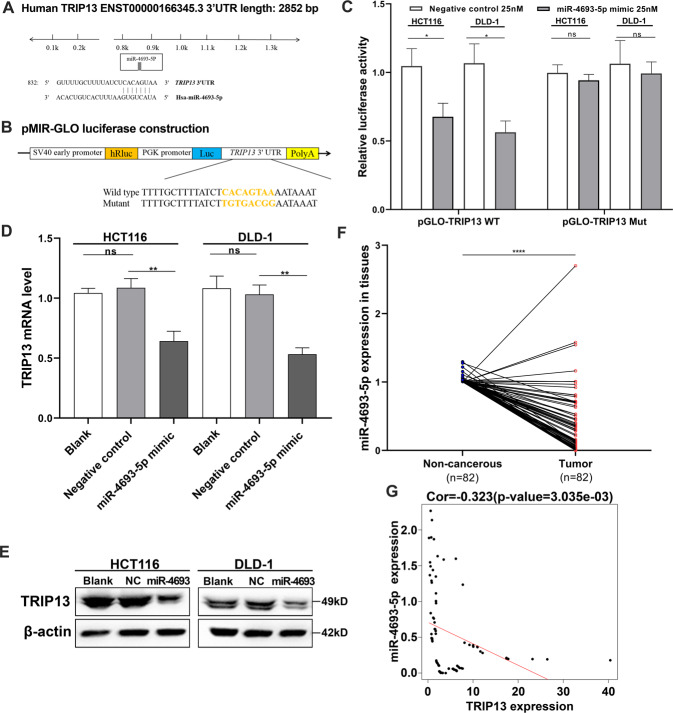
TRIP13 was a direct target of miR-4693-5p in CRC. **A** Human TRIP13 3′UTR binding site for miR-4693-5p. **B** The miR-4693-5p wild type binding sequence or its mutated form was inserted into C-terminal of the luciferase gene to generate pMIR-TRIP13-3′UTR or pMIR-TRIP13-mut-3′UTR, respectively. **C** miR-4693-5p targeted the wild-type but not the mutant 3′UTR of TRIP13. **D** Ectopic expression of miR-4693-5p downregulated TPIP13 mRNA expression in CRC cells. **E** miR-4693-5p decreased TRIP13 protein level in CRC cells, NC:negative control. **F** miR-4693-5p level was significantly decreased in CRC tumor. **G** Inverse association between miR-4693-5p and TRIP13 mRNA expression. *p < 0.05, **p < 0.01, ****p < 0.0001; *ns,* no significant difference.

Next, we explored how TRIP13 was upregulated in CRC cells. miRNA is a potent gene expression regulator. We speculated that there might be a specific miRNA involved in TRIP13 expression regulation. The bioinformatics algorithm, TargetScan, was used to predict that TRIP13 3′UTR contained a binding site for miR-4693-5p and miR-4693-5p might target it (Fig. 4A). The reporter vector, which consisted of the luciferase coding sequence and wild type or mutated sequence of TRIP13 3′UTR containing the predicted binding sites, was constructed to test the specific regulation of miR-4693-5p (Fig. 4B). Co-transfection experiments were performed in HCT116 and DLD-1 cells, and it was found that miR-4693-5p significantly decreased the Luc-TRIP13-3′UTR but not the Luc-TRIP13-mut 3′UTR luciferase activity (Fig. 4C). To further confirm that TRIP13 was a direct target of miR-4693-5p, TRIP13 mRNA and protein level were assessed after transfecting miR-4693-5p mimic or negative control into HCT116 and DLD-1 cells. Our data showed that TRIP13 mRNA and protein level was distinctly downregulated after transfection of miR-4693-5p mimic (Fig. 4D, E). The miR-4693-5p level in CRC tumor tissues was assessed. In CRC tumors, miR-4693-5p level decreased significantly (Fig. 4F) and was negatively correlated with TRIP13 expression (Fig. 4G).

### DCZ0415 suppressed CRC cell proliferation, invasion as well as migration, promoted cell apoptosis, and resulted in the arrest of cell cycle

**Fig. 5:**
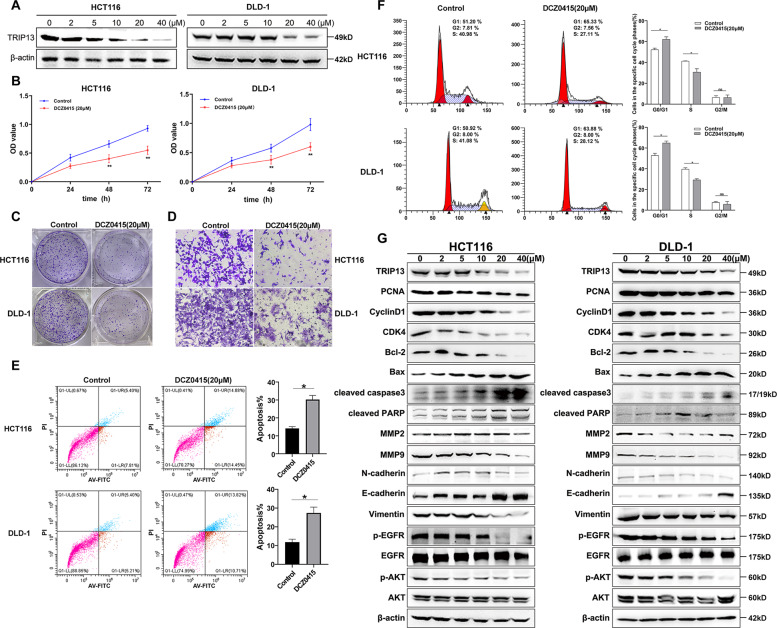
DCZ0415 suppressed CRC cell proliferation, invasion as well as migration, promoted cell apoptosis, and resulted in the arrest of cell cycle. **A** DCZ0415 decreased the expression of TRIP13 in CRC cells. **B** DCZ0415 inhibited cell proliferation of CRC cells. **C** DCZ0415 significantly reduced number of cell colonies. **D** DCZ0415 suppressed invasion and migration of CRC cells. **E** DCZ0415 increased apoptosis of CRC cells. **F** DCZ0415 induced G0/G1 phase arrest of CRC cells. **G** The protein level of PCNA, Bcl-2, Cdk4, Cyclin D1, MMP2, MMP9, Vimentin, N-cadherin, p-EGFR, and p-AKT was downregulated, whereas E-cadherin, Bax, cleaved PARP, and caspase3 level were upregulated. *p < 0.05, **p < 0.01; *ns,* no significant difference.

To verify that TRIP13 is a potential target for the treatment of CRC, the anti-tumor activity of DCZ0415, a small molecule inhibitor of TRIP13, was evaluated [24]. The optimal concentration of DCZ0415 to inhibit the protein expression of TRIP13 was also quantified. The results showed that the expression of TRIP13 significantly decreased after treatment with 20 µmol/L DCZ0415 for 48 h in both HCT116 and DLD-1 cells (Fig. 5A). The concentration of 20 µmol/L was used for the subsequent experiments.

According to the results of CCK8 and colony formation assays, the proliferation of cells significantly decreased following treatment with DCZ0415 (Fig. 5B, C). The transwell assay was performed to test whether DCZ0415 affected the migration of CRC cells. The results demonstrated that DCZ0415 significantly inhibited the CRC cells migration compared with the control group (Fig. 5D). Apoptosis and cell cycle progression was detected by flow cytometry following treatment with DCZ0415 for 48 h. DCZ0415 significantly induced apoptosis in CRC cells compared to control cells exposed to DMSO (Fig. 5E). Treatment-induced a significant accumulation in G0/G1 CRC cells (Fig. 5F). In addition, the protein level of PCNA, Bcl-2, CDK4, Cyclin D1, MMP2, MMP9, Vimentin, and N-cadherin was downregulated, whereas E-cadherin, BAX, cleaved PARP, and caspase3 level was upregulated (Fig. 5G). The expression of p-EGFR and p-AKT decreased in CRC cells after DCZ0415 treatment suggesting that the anti-tumor effect of DCZ0415 was involved in the EGFR/AKT signaling pathway (Fig. 5G). The above findings indicated that DCZ0415 suppressed CRC cell proliferation, invasion, and migration, promoted cell apoptosis, and resulted in the cessation of the cell cycle.

### In-vivo inhibition of tumor growth activity

**Fig. 6:**
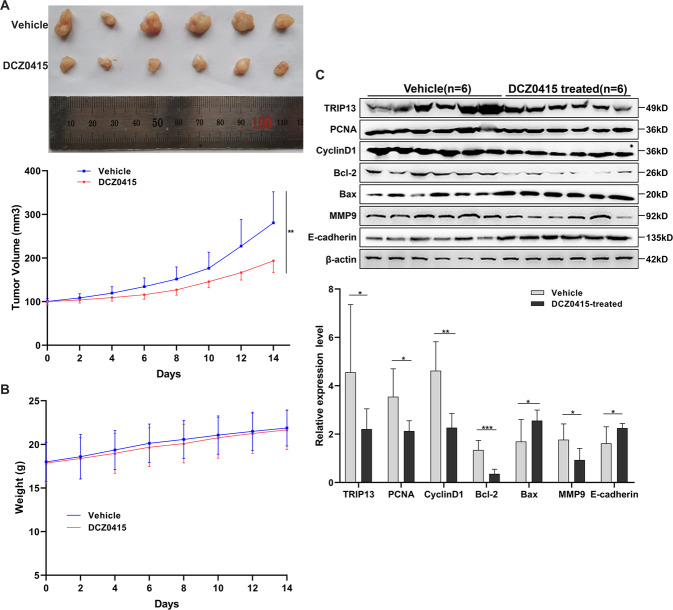
DCZ0415 inhibited HCT116 xenograft tumor growth in nude mice. **A** Effect of DCZ0415 on tumor volume. **B** Body weight in HCT116 human CRC xenograft tumor model after treatment. **C** DCZ0415-treated tumors showed similar changes in expression of TRIP13, PCNA, Cyclin D1, Bax, Bcl-2, MMP9, and E-cadherin as those in the DCZ0415-treated CRC cells. *p < 0.05, **p < 0.01, ***p < 0.001.

The CRC xenograft mouse model was employed to investigate the therapeutic potential of DCZ0415 in vivo. Compared to the control mice, the growth of tumors significantly reduced after the treatment with DCZ0415 (Fig. 6A). DCZ0415 was well tolerated because there was no significant difference in body weight between the DCZ0415-treated and control groups (Fig. 6B). Proteins were extracted from tumors from each group and subjected to Western blotting analysis to determine TRIP13, PCNA, Cyclin D1, BAX, Bcl-2, MMP9, and E-cadherin. DCZ0415-treated tumors showed similar changes in expression of these proteins as the DCZ0415-treated CRC cells (Fig. 6C). These results indicated that DCZ0415 had a therapeutic effect on CRC in-vivo.

## Discussion

It was anticipated to have 2.2 million new cases and 1.1 million deaths, causing the global burden of CRC to increase by 60% by 2030 [25]. The mortality of late-stage CRC is significantly higher than in the early stage because of the metastasis to secondary organs such as the liver [26]. CRC is more likely to be ignored by patients in the early stage, thus missing the best treatment time. The biomarkers of early-stage CRC, even adenoma diagnosis and treatment, play an integral part in CRC control. This study identified TRIP13 as a hub gene of CRC progression by bioinformatics analysis, and TRIP13 expression was verified in clinical specimens and various CRC cell lines. TRIP13 expression increased continuously from normal tissue to polyp/adenoma tissue to tumor tissue, suggesting the TRIP13 was related to the tumor progression and upregulated in early premalignant lesions. The relationship between TRIP13 and CRC patient survival was preliminarily analyzed, suggesting that TRIP13 is a potential biomarker for the treatment and prognosis of CRC, primarily early-stage CRC.

TRIP13, a hormone-dependent transcription factor, plays an essential role in DNA damage repair and division [27–29]. TRIP13 was first reported as an oncogene of head and neck cancer in 2014 [30], then other subsequent studies on cancers identified that TRIP13 might have oncogenic effects by promoting tumor cell proliferation, invasion, and metastasis [31–33]. It was reported that TRIP13 induced the degradation of Mad2, a part of the mitotic checkpoint complex and essential in the segregation of chromosomes [34, 35]. Mad2 degradation causes chromosome missegregation during mitosis, resulting in cancer progression and chemotherapy resistance [36]. TRIP13 was reported to promote non-homologous end joining (NHEJ), which was a repair method of DNA double-strand breaks and promoted chromosome instability as well as tumorigenesis [27, 30]. In hepatocellular carcinoma and bladder cancer cells, TRIP13 knockdown induced the increase of E-cadherin and the decreases of N-cadherin and Snail, suggesting that TRIP13 promotes metastasis *via* inducing the EMT [37, 38]. There are several studies on the relevance between TRIP13 and CRC. The oncogenic role of TRIP13 was confirmed in vitro and in vivo, and TRIP13 was related to the TNM tumor stage, CEA, and CA19–9 level in CRC patients [23, 39]. TRIP13 knockdown inhibits cell proliferation, colony formation, invasion, and cell motility, independent of the p53 and MS status of the cells [40]. TRIP13 might promote metastasis of CRC by interacting with the 14–3–3 protein superfamily member-YWHAZ, critical for cell cycle and EMT [23, 41]. TRIP13 interacts with FGFR4 and activates the EGFR/AKT pathway [40]. TRIP13 also regulates the β-catenin-dependent expression of Cyclin D1 and is involved in the WNT/β-catenin pathway [40]. However, TRIP13 as a therapeutic target for CRC needs further study through small molecule intervention.

DCZ0415 is a small molecule inhibitor designed to target TRIP13 [24]. Our studies employed CRC cells and xenograft models to demonstrate the anti-tumor activity of DCZ0415. DCZ0415 markedly suppressed CRC cell proliferation, migration, and tumor growth, promoted cell apoptosis, and resulted in the arrest of the cell cycle. After treatment with the DCZ0415, the expression of cell proliferation, apoptosis, cell cycle, and EMT-related proteins was affected. Our results showed that DCZ0415 reduced the expression of p-EGFR and p-AKT, suggesting that the anti-tumor effect of DCZ0415 was involved in the EGFR/AKT signaling pathway.

Meanwhile, the molecular mechanism of how TRIP13 expression is upregulated in CRC tumors is not entirely clear. Our study is the first to report that miR-4693-5p was significantly downregulated in CRC tissues and bound to the 3′ untranslated region (3′UTR) of TRIP13, downregulating TRIP13 expression. miRNAs regulated more than 30% of human genes, and the actions of miRNAs were pleiotropic [42, 43]. miRNAs affected cellular functions such as cell proliferation, malignant transformation, angiogenesis, inflammation [44]. miRNA dysregulation was involved in the early step of tumor formation [42, 45]. Based on the classical dichotomy of the oncogene-tumor suppressor, miRNAs could play carcinogenesis and anti-carcinoma roles in tumor development according to their relative expression level [44, 46]. There are several studies on miRNA regulation in CRC, such as miR-21, miR-92a, miR-135b, miR-106a, miR-143, miR-320e miR-224, miR-200c, miR-141, miR-30, miR-5000 [47–52]. Our results showed significant downregulation of miR-4693-5p in CRC tissues, suggesting it might act as a tumor suppressor.

In summary, TRIP13 expression increased continuously from adjacent mucosa to adenoma to early-stage tumor to late-stage tumor, and high expression of TRIP13 conferred a survival disadvantage to CRC patients. The underlying mechanism of TRIP13 expression upregulation in CRC tumors was the decrease of miR-4693-5p that targets TRIP13. DCZ0415, a small molecule inhibitor targeting TRIP13, induced anti-tumor activity in vitro and in vivo. DCZ0415 markedly suppressed CRC cell proliferation, migration, and tumor growth, promoted cell apoptosis, and resulted in the arrest of the cell cycle. Our research suggested that TRIP13 might play a crucial role in the CRC progression and assist in CRC treatment.

## Materials and methods

### Differentially expressed genes (DEGs) in early-stage CRC and construction of WGCNA

GSE9348 dataset comprising 80 samples (Tumors from 70 early-stage CRC patients and biopsies from 12 healthy controls) was used to obtain the DEGs between the early-stage CRC and the healthy controls. Three R language packages (“Affy,” “limma,” and “ggplot2”) were used to standardize the chip’s data, get and visualize the DEGs. With the characteristics of |log_2_fold change (FC) | ≥ 1 and adjusted *p*-value < 0.05, genes were considered differentially expressed. Then, the DEGs of GSE9348 were selected to apply WGCNA in GSE41258 data and get the co-expressed modules and hub genes related to the tumor progression. GSE41258 dataset included normal colons of 54 healthy controls, polyps of 49 patients, primary tumors of 185 patients, and metastasis tumors of 67 patients. According to the progression of the tumor, the trait was normal, polyp, primary tumor, and metastasis. Pearson’s correlation tests were used to apply the relevance analysis between module eigengene and trait. The candidate modules were selected when *p* < 0.05.

### Gene ontology (GO) and protein–protein interaction (PPI) enrichment analysis and hub genes screening of candidate module

To investigate the functional differences and involved biological processes of genes in the candidate module, the Metascape database (http://metascape.org/gp/index.html#/main/step1) was used to carry out the GO terms and pathway enrichment analysis. Terms with a minimum count of 3, enrichment factor >1.5, *p*-value < 0.01 were collected. According to the membership similarities, these terms were grouped into clusters. Online Database Search Tool for the Retrieval of Interacting Genes/Proteins (STRING, version 11.0; http://string-db.org/) was used to construct the PPI network of the candidate module genes. The parameter of interactions was set as medium confidence >0.4. Cytoscape (version 3.5.1) software was used to draw their interactions, and CytoHubba plug-in was used to screen the top 20 hub genes of the entire network. TRIP13 was selected as the hub gene.

### TRIP13 expression in GSE117606 dataset and survival analysis based on TCGA database

GSE117606 dataset, which analyzed adjacent mucosa from 65 patients, adenomas from 69 patients, and tumors from 74 patients, were used to verify the TRIP13 expression. The clinical parameters and survival data of 556 CRC patients from the TCGA database were downloaded to apply the preliminary study on the relevance between TRIP13 level and CRC patient survival. R language packages “survival” and “survminer” were used to plot the Kaplan–Meier survival curve and calculate the Log-rank *p*-value. According to the median value of gene expression, patients were divided into two groups.

### CRC clinical samples and cell lines

This study obtained primary CRC tumor and non-carcinoma samples (5 cm from tumor edge) from CRC patients (*n* = 82, aged 20–75 years) receiving surgery at Shenzhen Second People’s Hospital (Shenzhen, China). The CRC patients were initially diagnosed without family genetic history and had not received radiotherapy or chemotherapy before surgery. After resection, the samples were preserved in the RNAlater™ stabilization solution (Thermo Fisher Scientific, Waltham, MA, USA) immediately, followed by preservation under −80 °C until further use. The clinicopathological parameters of the sample are shown in Table 1. Human colon adenocarcinoma HCT116 cells and Human normal colorectal epithelial FHC cells were purchased from the ATCC (Manassas, VA, USA). The human colon adenocarcinoma cell lines, viz., HT-29, DLD-1, LS174T, Caco-2, LoVo, and SW480, were obtained from Cell Bank, China Academy of Sciences (Shanghai, China). Each cell line was recently authenticated by STR profiling and tested for mycoplasmacontamination, then cultivated in line with specific protocols.

**Table 1:** The clinicopathological parameters of 82 CRC patients.

Parameters	Number of patients (*N*%)
Gender	
Male	46 (56.1%)
Female	36 (43.9%)
Age	
<50	24 (29.27%)
≥50	58 (70.73%)
TNM stage	
I + II	37 (45.12%)
III + IV	45 (54.88%)
Lymph nodes metastasis	
N0	37 (45.12%)
N1 + N2	45 (54.88%)
Distant metastasis	
M0	70 (85.36%)
M1	12 (14.64%)

N0: No regional lymph node metastasis; N1: Metastasis in 1–3 regional lymph nodes; N2: Metastasis in 4 or more regional lymph nodes.

M0: No distant metastasis; M1: Distant metastasis.

### RNA extraction from tissues, cells, and qRT-PCR

TRIzol reagent (Thermo Fisher Scientific) and RNAiso for Small RNA (Takara) were used to extract total RNA and microRNA from the colorectal tumor tissues, non-cancerous tissues, and CRC cells, respectively. RNA (1 µg) was reverse transcribed using the BeyoRT™ II thesis kit with gDNA Eraser (Beyotime Bio Inc, China). small RNA (6 µg) was reverse-transcribed using the Mir-X miRNA First-Strand Synthesis Kit (Takara). The 7500 PCR system (Thermo Fisher Scientific) was adopted for qRT-PCR analysis, and the procedure was completed under the following conditions: 5 min at 95 °C; followed by 10 s at 95 °C and 35 s at 60 °C for 40 cycles (Takara Bio, Japan). The primer sequences used are shown in Table S1. GAPDH and snRNA U6 were used as the internal controls, and the 2^−△△Ct^ method was used to calculate the relative expression levels of TRIP13 and miR-4693-5p.

### Western blotting

Western blotting was conducted following specific protocols [53]. The radioimmunoprecipitation assay (RIPA) buffer was used to lyse CRC cells and tissues for 50 min on ice, followed by 15 min centrifugation of whole-cell lysates at 15,000 rpm at 4 °C. Bicinchoninic acid (BCA) assay was used to determine the protein concentration. Each sample was added with the loading buffer (Beyotime Bio Inc, China), followed by 5 min of denaturation at 95 °C. In the present work, the primary antibodies utilized included anti-TRIP13 (Abcam,Cat#ab204331), anti-PCNA (CST, Cat#2586S), anti-MMP2 (Proteintech, Cat#10373-2-AP), anti-MMP9 (Proteintech, Cat#10375-2-AP), anti-N-cadherin (CST, Cat#13116T), anti-E-cadherin (CST, Cat#3195T), anti-Vimentin (CST, Cat#5741S), anti-CyclinD1 (CST, Cat#55506S), anti-Cdk4 (CST, Cat#12790T), anti-Bcl-2 (CST, Cat#15071T), anti-Bax (CST, Cat#5023S), anti-cleaved PARP (CST, Cat#5625S), anti-cleaved caspase3 (CST, Cat#9664S), anti-p-EGFR (Try1068, CST, Cat#2236S), anti-EGFR (Abcam, Cat#ab52894), anti-p-AKT (Ser473, CST, Cat#4060S), anti-AKT (CST, Cat#4685S) and anti-β-actin (Beyotime Bio Inc, Cat#AA128). The enhanced chemiluminescence (ECL) detection system was employed to detect the protein-antibody complex. Protein level was quantified by the relative gray value using β-actin as a reference. Representative images of three independent experiments are shown in the figures.

### Vector construction and dual-luciferase reporter assay

The online tool TargetScan (www.targetscan.org) was used to find the potential binding sites of miR-4693-5p. We synthesized a sequence of 200 bp fragments containing the wild-type or mutant seed region of TRIP13, which was cloned into a pmirGLO luciferase vector. The sequence of 200 bp fragments is shown in Table S2. CRC cells were seeded in 96-well plates and co-transfected with miR-4693-5p mimic or negative control (25 nM) and pGLO vector (200 ng/well) by using lipofectamine 3000 (Invitrogen). Cells were harvested, and luciferase activities were analyzed (Promega, Madison, WI) at 48 h after transfection.

### Cell growth and colony formation assays

In order to verify that TRIP13 is a potential therapeutic target for CRC, we tested the anti-tumor effect of DCZ0415 (TOPSCIENCE, Shanghai, China), a small molecule inhibitor targeting TRIP13 by performing a series of experiments for functional assays. For colony formation, CRC cells (1 × 10^4^) were seeded into six-well plates, cultured for 12 h, and treated with or without DCZ0415 for 7 days. Subsequently, the viable colony number (>50 cells/colony) was estimated, and the colonies were stained thrice with Giemsa stain. Besides, cells were seeded in 96-well plates and treated with or without DCZ0415 for 24, 48, and 72 h. The Cell Counting Kit (CCK)−8 assays were performed to plot the cell proliferation curve.

### Transwell assay

CRC cells were cultured in a previously coated upper transwell chamber (Corning, USA), and then the medium containing 20% fetal bovine serum was added to the lower chamber. After 36 h, each transwell chamber was washed with PBS twice, and the non-invasive cells were gently removed using cotton swabs. The invasive cells were fixed with 100% methanol and stained with 0.25% crystal violet for over 15 min at room temperature. Each sample was read at five random positions in each image.

### Apoptosis and cell cycle analysis

The apoptosis and cell cycle analysis was performed using flow cytometry. Cells were seeded into six-well plates and treated with or without DCZ0415 for 48 h. According to the manufacturer’s instructions, apoptotic cells were measured with FITC AnnexinV Apoptosis Detection Kit (Yeasen, China). For cell cycle analysis, the collected cells were washed with PBS twice. After the supernatants were discarded, 500 µL of the 70% pre-cooled ethanol was added to fix cells overnight at 4 °C. After removing ethanol, a cold PBS solution was used to wash cells thrice. Then, 500 µL PI/RNase (Yeasen, China) staining working solution was added to stain cells for 30 min at 4 °C and observed.

### In vivo anti-tumor study

Human colorectal carcinoma xenograft was established in 12 male athymic Balb/c nude mice (5–6 weeks old, Guangdong Medical Laboratory Animal Center, China). HCT116 cells (5.0 × 10^6^/0.2 mL) were injected subcutaneously to the right flank of mice. The mice were randomly divided into the vehicle, and DCZ0415 treated groups with 6 mice in each group when tumor volume reached an average of ~100 mm^3^. Mice in each group were injected iv. with DCZ0415 (50 mg/kg) or vehicle control once every 2 d for two weeks. Subsequently, the mice were sacrificed, and the primary tumor was collected at the end of the experiment. Tumor volume was determined by caliper measurements (mm), using the formula of tumor volume (mm^3^) = (length × width^2^)/2.

### Statistical analysis

Statistical analysis was completed using GraphPad prism8 and SPSS 20.0. Each cell experiment was conducted thrice. The values were presented as mean ± SD from three independent experiments. The student’s t-test analyzed comparisons of two groups and the variance similar between the groups were analyzed by F test. Analysis of variance (ANOVA) was used for comparisons among multiple groups. The survival rate was calculated using the log-rank test, and comparisons were confirmed by the Kaplan–Meier method. Pearson’s correlation coefficient was used to analyze the correlation of the observed indicators. A difference of *p* < 0.05 (two-side) indicated statistical significance. *p < 0.05, **p < 0.01, ***p < 0.001, ****p < 0.0001, ns: no significant difference.

## Supplementary information

supplementary figure legends

Figure S1

Figure S2

Figure S3

Figure S4

Table S1

Table S2

Author contribution form

## Data Availability

The data supporting the results of this study are available in GEO and TCGA database. This data was derived from the following public resources: https://www.ncbi.nlm.nih.gov/gds, http://cancergenome.nih.gov, http://www.cbioportal.org, and https://portal.gdc.cancer.gov.
