# Supplementary material for: TRIP13, identified as a hub gene of tumor progression, is the target of microRNA-4693-5p and a potential therapeutic target for colorectal cancer
Source: Cell Death Discov. 2022 Jan 24;8:35. doi: 10.1038/s41420-022-00824-w (PMC8786872; doi:10.1038/s41420-022-00824-w)
Supplement: Supplementary file 1 — supplementary figure legends [file 41420_2022_824_MOESM1_ESM.doc]

**Supplementary Figure Legends**

Fig. S1. The genes enrichment and eigengene adjacency heatmap.

1. The scatter plot between the blue module membership and the gene significance for polyp.
2. The scatter plot between the blue module membership and the gene significance for primary tumor.
3. Heatmap of the adjacencies of modules. Red represented high adjacency and blue represented low adjacency.

Fig. S2. Gene ontology analysis and significant enrichment of the blue module genes.

1. Network of functional and pathway enrichment colored by cluster ID. Different colors in the map represent different functional groups.
2. Network showing functional and pathway correlations colored by p-value. The darker color means the more genes were enriched in this pathway or biological process.
3. The heatmap of the involved biological processes of 20 hub genes.

Fig. S3. The protein level of TRIP13 in tissues.

The protein level of TRIP13 was drastically increased in CRC tumors compared to non-cancerous tissues. N:Non-cancerous, T:Tumor.

Fig. S4. The protein level of TRIP13 in CRC cells.

The protein level of TRIP13 was significantly increased in CRC cells compared to FHC cells.
