# Supplementary figures and images for: TRIP13, identified as a hub gene of tumor progression, is the target of microRNA-4693-5p and a potential therapeutic target for colorectal cancer
Source: Cell Death Discov. 2022 Jan 24;8:35. doi: 10.1038/s41420-022-00824-w (PMC8786872; doi:10.1038/s41420-022-00824-w)

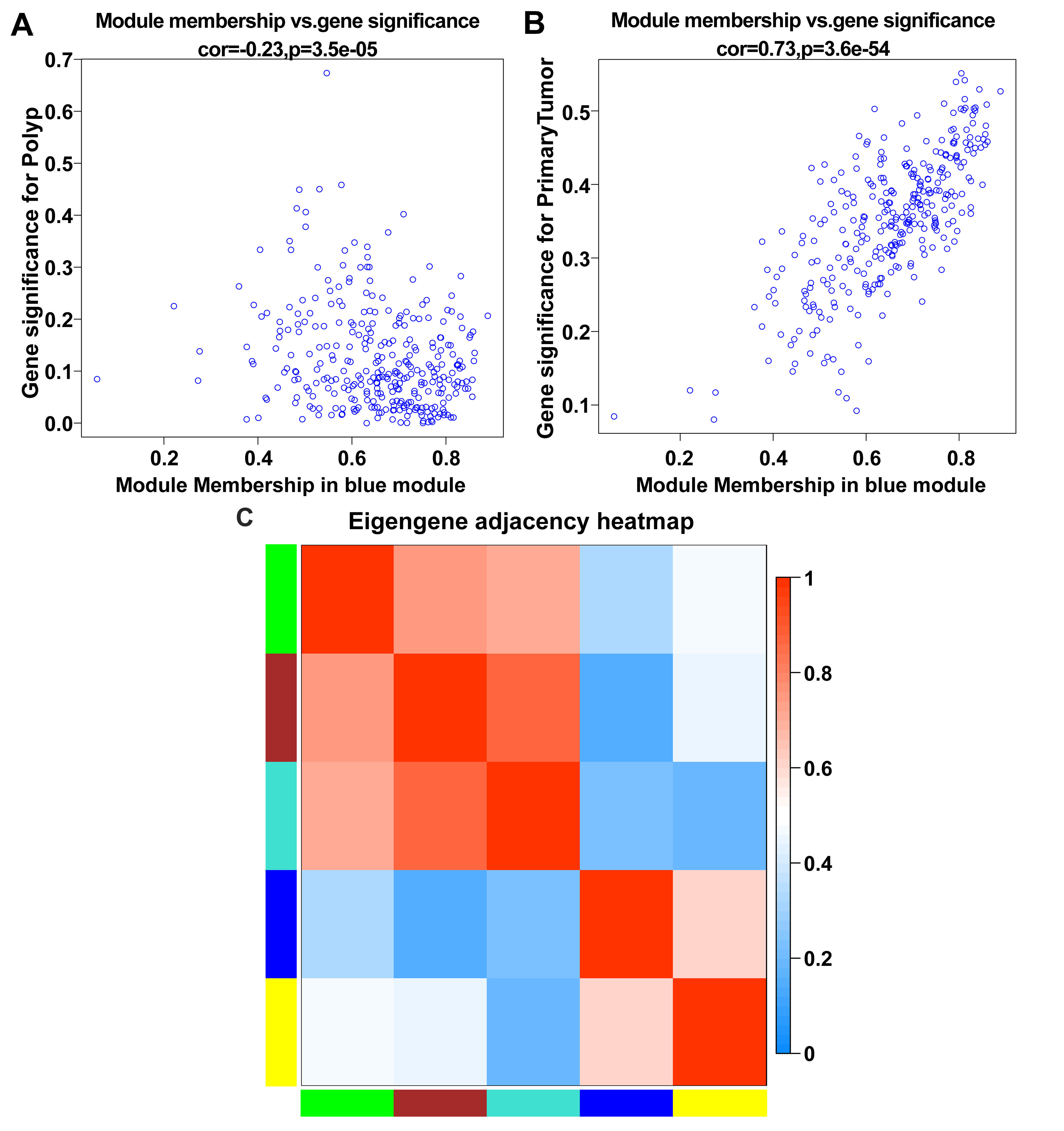

Supplement: Supplementary file 2 — Figure S1 [file 41420_2022_824_MOESM2_ESM.tif]

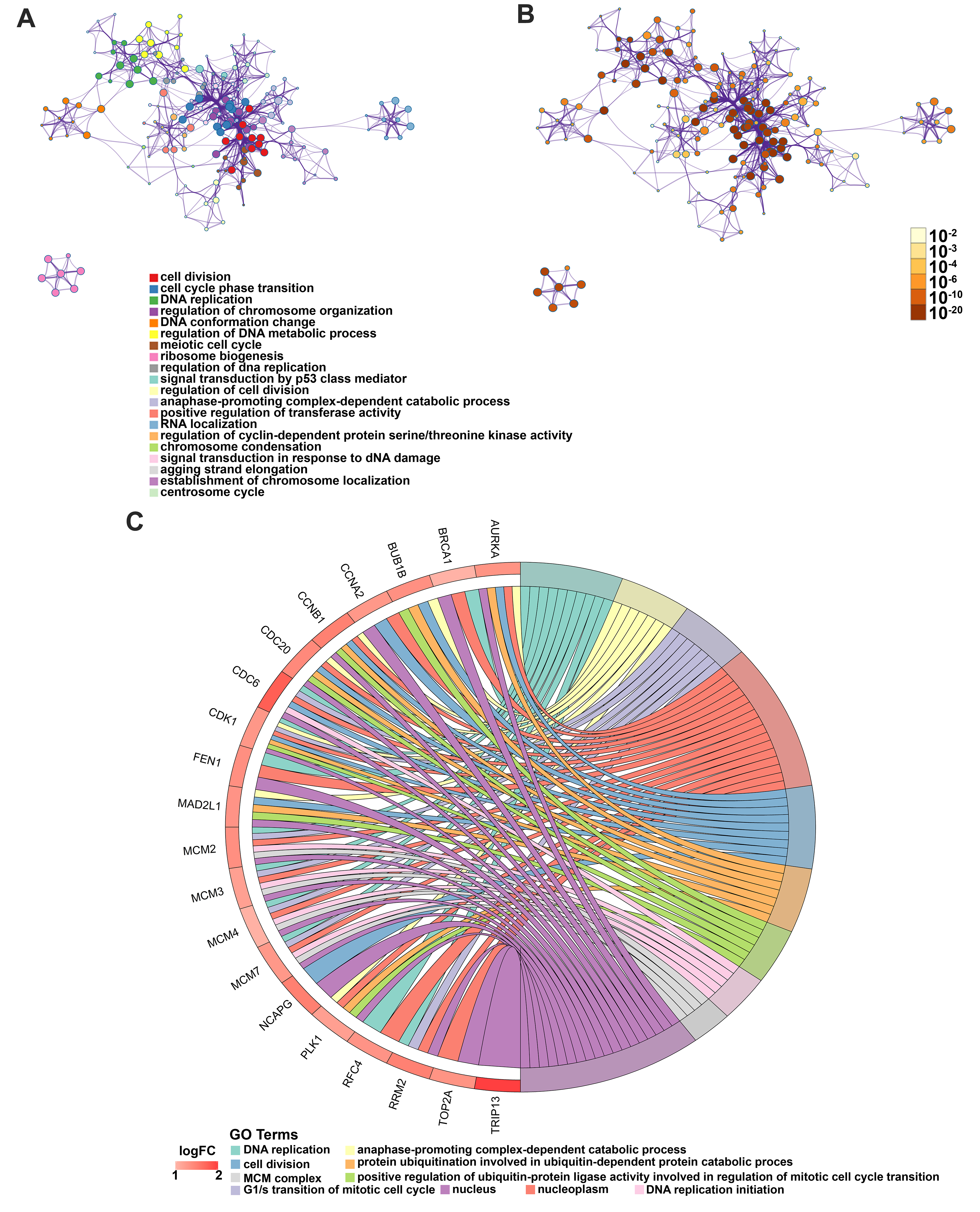

Supplement: Supplementary file 3 — Figure S2 [file 41420_2022_824_MOESM3_ESM.tif]

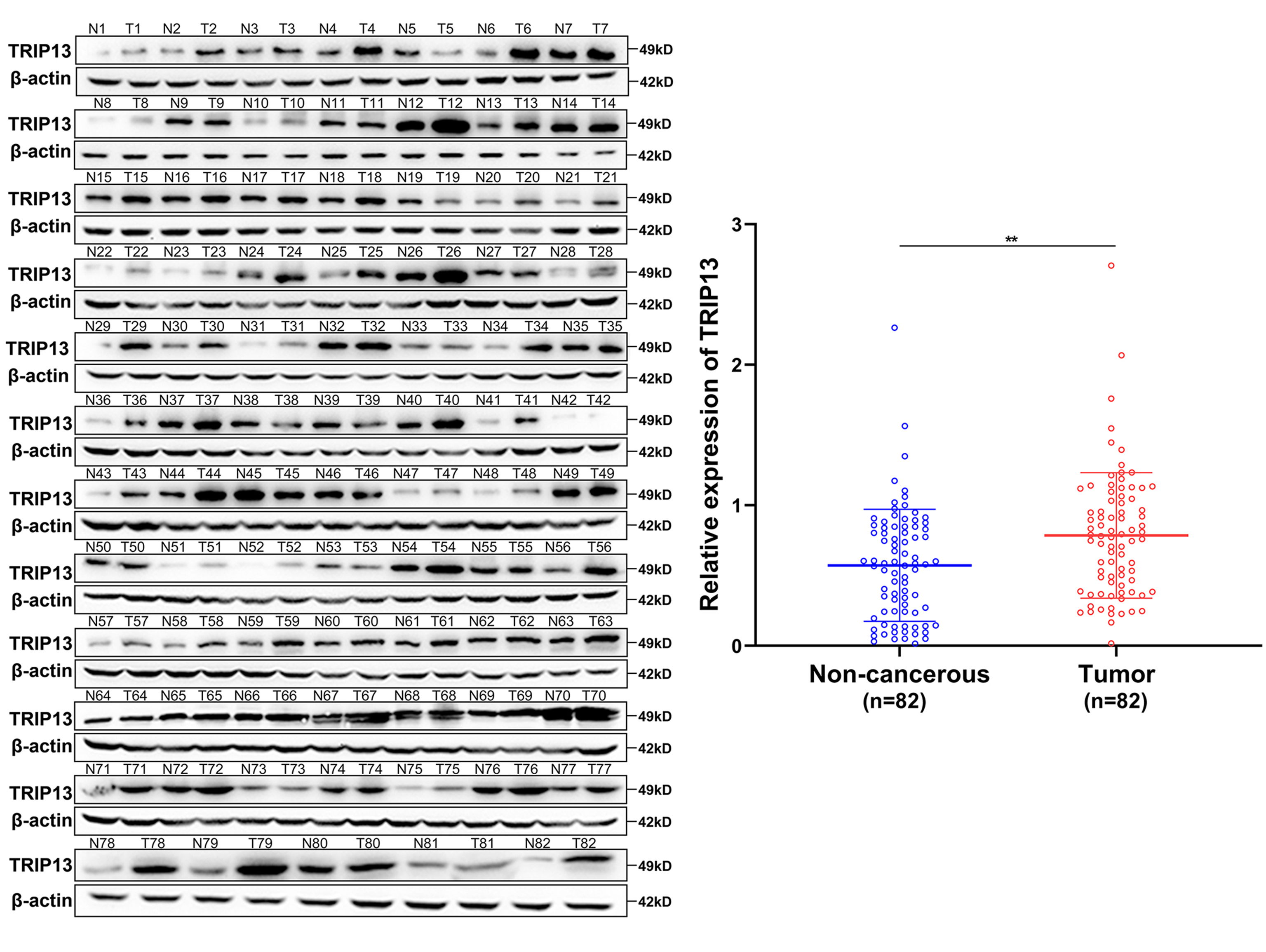

Supplement: Supplementary file 4 — Figure S3 [file 41420_2022_824_MOESM4_ESM.tif]

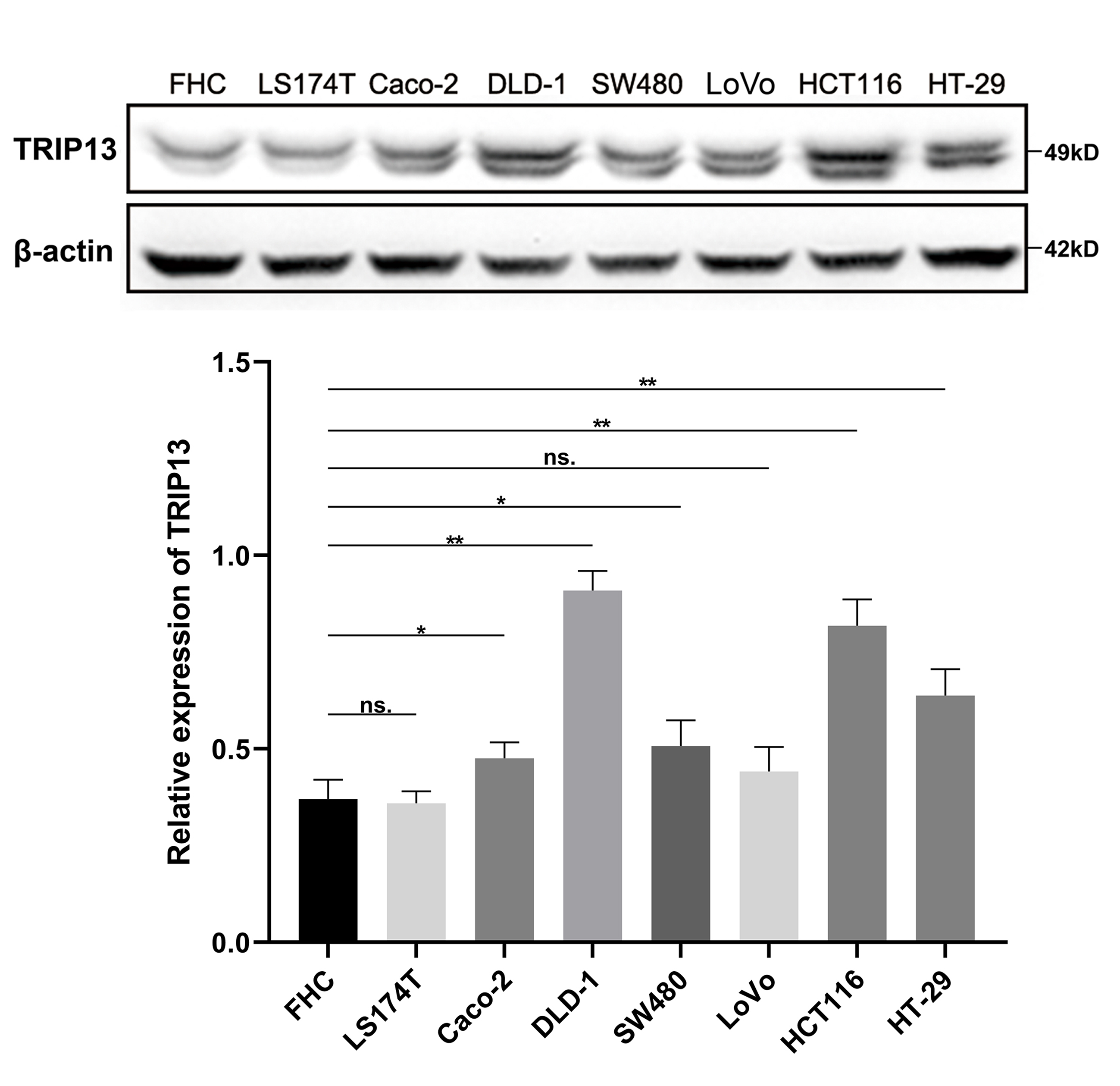

Supplement: Supplementary file 5 — Figure S4 [file 41420_2022_824_MOESM5_ESM.tif]
