# Supplementary material for: TRIP13, identified as a hub gene of tumor progression, is the target of microRNA-4693-5p and a potential therapeutic target for colorectal cancer
Source: Cell Death Discov. 2022 Jan 24;8:35. doi: 10.1038/s41420-022-00824-w (PMC8786872; doi:10.1038/s41420-022-00824-w)
Supplement: Supplementary file 6 — Table S1 [file 41420_2022_824_MOESM6_ESM.doc]

**Table S1. Primer sequences used for qRT-PCR**

| **Gene** | **Sequence** |
| --- | --- |
| GAPDH-F | GAGTCAACGGATTTGGTCGT |
| GAPDH-R | TTGATTTTGGAGGGATCTCG |
| TRIP13-F | GGGTCCTGAGAAAACTCCCC |
| TRIP13-R | GGTGGCTTTCTAGCTTGCAGT |
| snRNA U6-F | CTCGCTTCGGCAGCACA |
| snRNA U6-R | AACGCTTCACGAATTTGCGT |
| miR-4693-5p-F | ATACTGTGAATTTCACTGTCACA |
| miR-4693-5p-R | Universal primer in Mir-X miRNA First-Strand Synthesis Kit of Takara |
