# Supplementary material for: TRIP13, identified as a hub gene of tumor progression, is the target of microRNA-4693-5p and a potential therapeutic target for colorectal cancer
Source: Cell Death Discov. 2022 Jan 24;8:35. doi: 10.1038/s41420-022-00824-w (PMC8786872; doi:10.1038/s41420-022-00824-w)
Supplement: Supplementary file 7 — Table S2 [file 41420_2022_824_MOESM7_ESM.doc]

**Table S2. The sequence containing the wild-type or mutant seed region of TRIP13**

| **Types** | **Sequence** |
| --- | --- |
| wild type of TRIP13 | 5’-GGGATGTTTCTGCCCACGGTTTTGTTTGTGCAATAACGTTATCACATTTCTAATGAGGATTCACATTAATATAATATAAAATAAATAGGTCAGTTACTGGTCTCTTTCTCCGAATGTTATGTTTTGCTTTTATCTTGTGACGGAATAAATATAATTAATGGTTTGCATGTGAAATTCACTTTTGAAAGAACATGTTACCTTACCTTTTGT-3’ |
| mutant type of TRIP13 | 5’-GGGATGTTTCTGCCCACGGTTTTGTTTGTGCAATAACGTTATCACATTTCTAATGAGGATTCACATTAATATAATATAAAATAAATAGGTCAGTTACTGGTCTCTTTCTCCGAATGTTATGTTTTGCTTTTATCTCACAGTAAAATAAATATAATTAATGGTTTGCATGTGAAATTCACTTTTGAAAGAACATGTTACCTTACCTTTTG-3’ |
