# Supplementary material for: TRIP13, identified as a hub gene of tumor progression, is the target of microRNA-4693-5p and a potential therapeutic target for colorectal cancer
Source: Cell Death Discov. 2022 Jan 24;8:35. doi: 10.1038/s41420-022-00824-w (PMC8786872; doi:10.1038/s41420-022-00824-w)
Supplement: Supplementary file 8 — Author contribution form [file 41420_2022_824_MOESM8_ESM.pdf]

# DECLARATION OF CONTRIBUTIONS TO ARTICLE **ADMC**

| Manuscript Number: | Journal Name: | |
|---|---|---|
| CDDISCOVERY-21-2089 | *Cell Death Discovery* | (the 'Journal') |

Proposed Title of the Contribution:

TRIP13, identified as a hub gene of tumor progression, is the target of microRNA-4693-5p and as a potential therapeutic target for colorectal cancer (the 'Contribution)

Author(s):

Yan Chen, Danqi Chen,Ying Qin, Cheng Qiu, Yaoyao Zhou, Mengmeng Dai,Lulu Li,Qinsheng Sun,Yuyang Jiang (the 'Authors')

For all *CDDiscovery* articles, each person named as an author in the published version must be able to show he or she has contributed substantially to the article.

Authorship credit should be based on 1) substantial contributions to conception and design, acquisition of data, or analysis and interpretation of data; 2) drafting the article or revising it critically for important intellectual content; and 3) final approval of the version to be published. Authors should meet conditions 1, 2 and 3.

Any person who cannot be shown to have made a substantial contribution to the article cannot be listed as an author in the final version. The name of any person who is deemed to have made a minor contribution can, however, appear in the Acknowledgments section of the article.

Please complete the table below to indicate the contributions of all named authors to the manuscript.

| Author Full Name: | Specification of Contribution to the Manuscript: |
|---|---|
| Yan Chen | YC was the principle investigator. YC conducted the majority of the molecular and cellular experiments. YC was a major contributor in writing the manuscript. |
| Danqi Chen | DQ C conducted a part of the molecular and cellular experiments. |
| Ying Qin | YQ collected the clinical specimens. |
| Cheng Qiu | CQ performed the animal experiments. |
| Yaoyao Zhou | YY Z helped perform the molecular and cellular experiments. |
| Mengmeng Dai | MM D helped perform the animal experiments and helped write the manuscript. |
| Lulu Li | LLL helped conduct data management and bioinformatics analysis. |
| Qinsheng Sun | QS S conducted statistical analysis. |
| Yuyang Jiang | YYJ conceived the idea for the paper and revised the manuscript critically. |
| | |
| | |
| | |
| | |

Please complete the table below to indicate the contributions of all named authors to the figures.

Figure 1:

In Figure 1, LL L generated the data and prepared panel A, YC conducted the bioinformatics analysis and assembled the figure.

Figure 2:

In Figure 2, LL L generated the data and conducted gene ontology (GO) and protein-protein Interaction (PPI) enrichment analysis , YC assembled the figure.

Figure 3:

In Figure 3, YQ collected the clinical specimens, QS S generated the data and prepared panel A and D, YC generated the PCR data and prepared panel B, MM D generated the PCR data and prepared panel C, YC assembled the figure.

Figure 4:

In Figure 4, YC conducted the dual-luciferase reporter assay , generated the PCR data and labelled the image, YY Z conducted the western blotting, QS S generated the data and prepared panel G, YC assembled the figure.

Figure 5:

In Figure 5, YC conducted the western blotting, generated the data and prepared panel A and G, YC conducted cell growth and colony formation assays, DQ C conducted transwell assay, apoptosis and cell cycle analysis, YC assembled the figure.

Figure 6:

In Figure 6, CQ and MM D conducted animal experiments, YY Z conducted the western blotting, generated the data and prepared panel C, YC assembled the figure.

| Signed for and on behalf of the Author(s): | Print Name: | Date: |
| --- | --- | --- |
| [signature] | Yuyang Jiang | 2021.7.15 |
